# Phytolith data in peat profile over the past 1300 years in the Xishan Mountains, Jiangxi Province, China

**DOI:** 10.1016/j.dib.2019.103981

**Published:** 2019-06-14

**Authors:** Xin-Rong Zhang, Yu Du, Chun-Mei Ma, Shuai-Fei Ping, Chong Feng

**Affiliations:** a College of Earth Sciences, Jilin University, Changchun, 130061, China; b Key-Lab for Evolution of Past Life and Environment in Northeast Asia, Ministry of Education, China; c School of Geography and Ocean Science, Nanjing University, Nanjing, 210023, China; d Faculty of Geographical Science, Beijing Normal University, Beijing, 100875, China

**Keywords:** Phytolith morphotypes, Microscopic observation, Description, Classification

## Abstract

Phytoliths are microscopic siliceous particles formed in the plants and preserved in the sediments after the plant death and decay. Phytolith formation is controlled by the plant genes and growing environments. As such, phytolith assemblages have been widely used in ancient plant composition analysis, paleoclimate reconstruction, and paleoenvironment reconstruction. For the effective utilization, phytolith description, nomenclature and classification are the most important. This article presents detailed original phytolith data from a peat profile (28°44′55.33″N, 115°39′59.80″N), which is related to the research article of “Climatic controls on peat swamp formation and evolution since 1300 year BP as recorded by phytoliths in the Xishan Mountains, Jiangxi Province, China” [1]. After extracted from peat, the phytoliths were observed under 400 × light microscope, described and nominated according to ICPN1.0 [2], and classified and counted more than 400 particles for each peat sample. 314 microscopic slides were observed and fifty types of phytolith were classified for the peat profile, including woody phytoliths, shrub phytoliths, herbaceous phytoliths and other unidentified morph types. All these provide basic information for paleo-researches.

**Table:** Specifications Table

Subject area	*Quaternary Geology*
More specific subject area	*Paleo-climatology*
Type of data	*Table Figure*
How data was acquired	*Light microscope observation*
Data format	*Raw*
Experimental factors	*Acid oxidation, Heavy liquid separation, Microscopic identification*
Experimental features	*Separation over* 72hrs *before using the 2.3g/cm3 heavy liquid; identification under 400* × *microscope, more than 350 grains counted for each sample*
Data source location	*Jilin University, China*
Data accessibility	*Data is available with this article*
Related research article	*Xin-Rong Zhang, Yu Du, Chun-Mei Ma, Shuai-Fei Ping, Chong Feng, An-ning Cui*[*1*]*, Climatic controls on peat swamp formation and evolution since 1300 year BP as recorded by phytoliths in the Xishan Mountains, Jiangxi Province, China, Palaeogeography, Palaeoclimatolgy, Palaeoecology*

**Table:** 

**Value of the data.** • The data can be used to compare the phytolith characteristics between different species of common plants in the mountainous area of humid subtropics zone in SE China. • Phytolith dry-wet index and cold-warm index calculated according to the descriptions can help identifying the hydrothermal environment changes during the peat development. • The phytolith data are essential for vegetation succession and the paleo-climate research. • It can be compared with the palynological study for more detailed paleo-researches.

## 1 Data

This article presents detailed original phytolith data from a peat profile (28°44′55.33″N, 115°39′59.80″N). It is related to the research article of “Climatic controls on peat swamp formation and evolution since 1300 year BP as recorded by phytoliths in the Xishan Mountains, Jiangxi Province, China” [1]. 157 peat samples were collected from the surface to the bottom of this profile. Each sample was 0.5cm thick and about 1.0g of dry weight. Samples were oxidized for 72hrs and separated by 2.3g/cm^3^ heavy liquid for pure phytolith concentration. 314 slides were made for phytolith microscopic observation.

On the basis of identification and counting under 400 × light microscope, more than 400 phytolith grains with diameters of >5 μm were counted in each two 1 cm^2^ microscope slides. There are fifty types of phytoliths, including Pteridophyta, conifers, broad-leaved, Cyperaceae, Bambusoideae, Chloridoideae, Panicoideae, Pooideae, Oryzoideae, and other “unidentified morphotypes”.

**Fig. 1:**
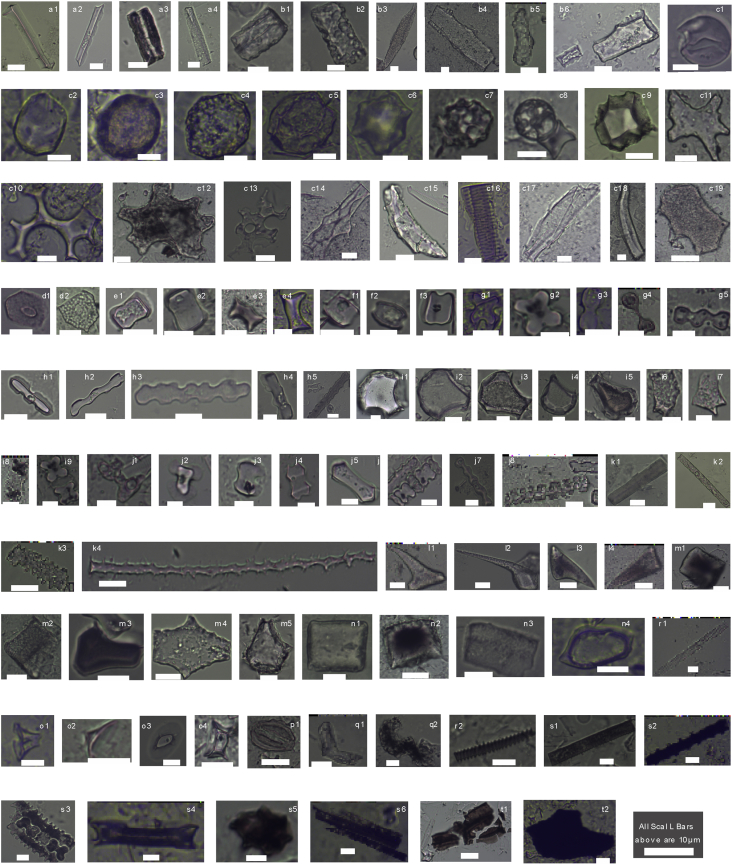
**Major phytolith micrographs and charred grains recovered from studied core.** a1-a4, from epidermal cells of *Pteridophyta*; b1-b6, from conifers; c1-c19, from broad-leaved trees; d1-2 polyhedron with papillae from sclerendhyma of *Cyperaceae*; e1-4, long saddles from epidermal cells of *Bambusoide*; f1-2 square saddle from epidermal cell of *Chloridoideae*; g1-5 cross from short epidermal cells of *Panicoideae;* h1-5 trapiziform from epidermal cells of *Pooideae*; i1-9 *Oryzoideae* phytolith; j1-8, k1-4, l1-4, m1-5, n1-4, o1-4, p1, q1-2, r1-2 are morphologies unidentified to genus; a3, c3, c12, c16, e3, g4, i5, i8, l3-4, m1, m3, n2, q2, r2, s1-6 are burned phytoliths, t1-2 are charcoal plates or grains extracted together with phytolith.

All these morphotypes were described according to ICPN1.0 [2] and classical literature [3] in Fig. 1, and Table .1. The calculated absolute concentrations of each sample can be found in the data link of the research article [1].

**Table 1:** Phytolith morphotype discriptions for peat profile.

Major plant	Morphotype	Anatomical origin	Fig.
Pteridophyta	Triangular prism with acute angle bottom	Epidermal cell	a1
	Triangular prism with sinuate edge		a2
	Elongate with two paralleled undulating ridges		a3
	Triangular prism with cancave edges and surfaces		a4
Conifers	Blocky polyhedral and cubic with clear ridge	Endodermal cell	b1
	Blocky scrobiculate	Blocky polyhedrons transfusion cell	b2
	Elongate irregular	Intercellular space	b3
	Tabular elongate cavate	Epidermal cells	b4
	Parallelepipedal contored		b5
	Tabular elongate dendric and blocky polyhedral	Transfusion traxheid	b6
Broad-leaved	Globular psilate	Epidermal cell	c1-2
	Globular granulate		c3-5
	Globular echinate		c6
	Globular cavate		c7-8
	Globular multifaceted		c9
	Stellate irregular jigsaw		c10-13
	Polyhedral facetate	Sclerenchyma	c14-15
	Elongate decorated	Tracheary tissue	c16-17
	Cylindrical sclereid		c18
	Tabular polygon	Epidermal cell	c19
Cyperaceae	Polyhedron with papillae	Sclerenchyma	d1-2
Bambusoideae	Long s ADdle or callpsed s ADdle	Epidermal cell from leaf/Culm/inflor	e1-4
Chloridoideae	Square s ADdle	Epidermal cell from leaf/Culm/inflor	f1-2
Panicoideae	Cross	Short epidermal cell	g1-2
	Bilobate	Short epidemal cell	g3-4
	Cylindrical polylobate	Epidermal cell	g5
Pooideae	Trapiziform bilobate	Epidermal cell	h1
	Trapeziform polylobate		h2-3
	Trapeziform sinuate		h4-5
Oryzoideae	Cuneiform bulliform cell	Leaf epidermal	i1-5
	Double peaked glume cell	Husk cell	i6-7
	Bilobate	Leaf epidermal	i8-9
Unidentified morphotypes	Bilobate	Epidermal cell	j1-6
	Polylobate		j7
	Elongate castelate	Epidermal long cell	j8
	Elongate smooth		k1
	Elongate coarse		k2
	Elongate echinate		k3-4
	Haircell	Hair cell	l1-2
	Scutiform		l3
	Lanceolate		l4
	Parallepipedal bulliform	Bulliform cell	m1-2
	Cuneiform bulliform		m3-5
	Square		n1-2
	Rectangle		n3
	Ovate		n4
	Conical hollow with flat top	Hair or trichome base	o1
	Conical hollow with cone top		o2
	Rondel		o3
	Pyramidal		o4
	Stoma cell	Stoma cell	p1
	Unciform	Traiched cell	q1-2
	Rugose elongate	Vascular cell or tissue	r1-2
Charred phytolith	Burned phytolith	a3,c3,c12,c16,e3,g4,i5,i8,l3-4,m1,m3,n2,q2,r2, s1-6	
Charcoal	Charcoal plates or grains	Residues	t1-2

## 2 Experimental design, materials and methods

### 2.1 Materials

Phytoliths were extracted from the peat samples of a 340cm depth peat profile. Each sample quantity is about 1 cm^3^. The sampling interval is every other 1 cm through the core.

### 2.2 Experimental design

(1) These sub-samples were dried at 60 °C and then weighed.
(2) The dried samples were initially disaggregated by being gently stirred in distilled water overnight.
(3) The samples were then heated for 1 hr in 250 ml beakers with 10 ml of 10% hydrochloric acid (HCl) and 75 mL of 68% nitric acid (HNO_3_).
(4) The samples were left to gravity settle for 4 hr, after the upper part was clean.
(5) The solution was then rinsed five times with distilled water until the pH was neutral.
(6) Dipersed Lycopodium spore tablet using diluted HCl (10%) and rinsed it to neutral.
(7) The samples were then transferred to 50 ml centrifuge tubes and mixed with 15 ml of 2.3 g/mL heavy liquid (ZnI_2_), and centrifuged three times for 10 min at 1500 rpm.
(8) Mixed the dispersed Lycopodium spores in the phytolith part.
(9) Transferred the mixed phytolith and Lycopodium to 5 ml vials for storage.
(10) Made slides with Canadian gum and prepare an optical microscope with 40 × objective lens and 10 × eye lens.

### 2.3 Methods

The absolute concentration of each type of phytolith was calculated using the following formula, on the basis of the number of added Lycopodium spores and the starting dry weight of each sample.

Phytolith concentration = [(counted phytolith number × Lycopodium number in a tablet)/counted Lycopodium number/dry weight of sample] (unit is × 10000grains/gram)

### 2.4 Dataset description

Pteridophyta phytoliths from the studied core are mainly triangular with acute angle bases (Fig. 1; a1), triangular with sinuate edges (Fig. 1; a2), elongate with two parallel undulating ridges (Fig. 1; a3), or triangular with concave edges and surfaces (Fig. 1; a4) [4], [5], [6].

The coniferous phytoliths [7], [8], [9] have blocky polyhedral and cubic shapes with clear ridges (Fig. 1; b1), blocky shapes with scrobiculate surfaces (Fig. 1; b2), elongate irregular shapes (Fig. 1; b3), tabular elongate cavate shapes (Fig. 1; b4), parallelepipedal contoured shapes (Fig. 1; b5), tabular elongate dendritic shapes, and blocky polyhedral (Fig. 1; b6) forms.

Broad-leaved plant phytoliths [10], [11], from this coremainly have globular psilate (Fig. 1; c1–2), globular granulate (Fig. 1; c3–5), globular echinate (Fig. 1; c6), globular cavate (Fig. 1; c7–8), globular multifaceted (Fig. 1; c9), stellate irregular jigsaw (Fig. 1; c10–13), polyhedral facetate (Fig. 1; c14–15), elongate decorated (Fig. 1; c16–17), cylindrical sclereid (Fig. 1; c18), and tabular polygons (Fig. 1; c19).

Cyperaceae phytoliths are mainly polyhedra with papillae, which are from sclerenchyma [12] (Fig. 1; d1–2). Bambusoideae phytoliths mainly have long saddle or collapsed saddle shapes, and originate from epidermal cells from leaves, stalks, and flowers [12], [13] (Fig. 1; e1–4). Chloridoideae phytoliths mainly have square saddle shapes and are from epidermal cells [3], [13] (Fig. 1; f1–2). Panicoideae phytoliths typically have cross (Fig. 1; g1–2), bilobate (Fig. 1; g3–4), and cylindrical polylobate (Fig. 1; g5) forms, which are also derived from epidermal cells [2], [12]. Pooideae phytoliths in this core are mainly epidermal cell phytoliths, including trapeziform bilobate (Fig. 1; h1), trapeziform polylobate (Fig. 1; h2–3), and trapeziform sinuate (Fig. 1; h4–5) forms [2], [3], [12], [13], [14]. Oryzoideae phytoliths [15] have cuneiform bulliform cell (Fig. 1; i1–5), double peaked glume cell (Fig. 1; i6–7), and bilobate forms.

The unidentified morphotypes types mainly include: (1) short cell bilobate (Fig. 1; j1–6) and polylobate (Fig. 1; j7) forms; (2) long epidermal cells with elongate castelate (Fig. 1; j8), elongate smooth (Fig. 1; k1), elongate coarse (Fig. 1; k2), and elongate echinate (Fig. 1; k3) forms; (3) Acicular and unciform hair cells (Fig. 1; l1–4); (4) parallelepipedal bulliform cells (Fig. 1; m1–2; Fig. 1; n1–2; Fig. 1; n3), cuneiform bulliform cells (Fig. 1; m3–5), ovate (Fig. 1; n4), conical hollow with flat top (Fig. 1; o1), conical hollow with cone top (Fig. 1; o2), rondel (Fig. 1; o3), pyramidal (Fig. 1; o4), stoma cell (Fig. 1; p1), and unciforms (Fig. 1; q1–2).
